# Prognostic nomogram for overall survival in breast invasive micropapillary carcinoma integrating LODDS and treatment factors: A SEER-based study

**DOI:** 10.17305/bb.2026.13884

**Published:** 2026-03-11

**Authors:** Ziqiang Wang, Hao Zhang, Haojie Zhang

**Affiliations:** 1 Department of Breast Surgery, Binzhou Medical University Hospital, Shandong, China; 2 Department of Thyroid Surgery, Binzhou Medical University Hospital, Shandong, China

**Keywords:** Invasive micropapillary carcinoma, SEER, nomogram, LODDS

## Abstract

Invasive micropapillary carcinoma (IMPC) of the breast is a rare and aggressive histologic subtype characterized by frequent lymph node metastasis and poor prognosis. The conventional American Joint Committee on Cancer (AJCC) tumor-node-metastasis (TNM) staging system does not account for treatment modalities or advanced nodal metrics such as the log odds of positive lymph nodes (LODDS), which may limit prognostic accuracy. This study aimed to develop and internally validate a nomogram integrating clinicopathologic characteristics, treatment variables, and LODDS to predict overall survival (OS) in breast IMPC. Clinicopathologic and survival data from 1105 patients diagnosed between 2010 and 2015 were obtained from the Surveillance, Epidemiology, and End Results (SEER) database. The entire cohort was used for model development, with bootstrap resampling for internal validation. Least absolute shrinkage and selection operator (LASSO) regression and multivariable Cox analysis were used for variable selection and nomogram construction. Model performance was assessed using the optimism-corrected concordance index (C-index), calibration plots, time-dependent area under the receiver operating characteristic curve (AUC), decision curve analysis (DCA), and clinical impact curves (CICs), while incremental value over the AJCC TNM system was evaluated by net reclassification improvement (NRI) and integrated discrimination improvement (IDI). Ten prognostic factors were retained in the nomogram: age, tumor size, LODDS, marital status, tumor grade, M stage, rural/urban residence, molecular subtype, radiotherapy, and chemotherapy. The nomogram showed superior discrimination to TNM staging, with better optimism-corrected C-index and 3-, 5-, and 10-year AUCs (all *P <* 0.05), significant improvements in NRI and IDI (all *P <* 0.001), excellent calibration, and greater net clinical benefit on DCA and CICs. Exploratory risk stratification identified high- and low-risk groups with significantly different survival outcomes (log-rank *P <* 0.001). This nomogram may improve prognostic assessment in breast IMPC, although the risk stratification remains exploratory and requires external validation before clinical application.

## Introduction

Invasive micropapillary carcinoma (IMPC) is a rare malignant tumor subtype distinguished by unique histopathological features and aggressive biological behavior. First identified in breast cancer by Siriaunkgul and Tavassoli in 1993, IMPC has since been recognized in various other organs, including the bladder, lung, colon, and stomach [1]. Although IMPC comprises a small percentage of breast cancers (approximately 2%–8%), its clinical significance should not be overlooked [2, 3]. The occurrence of pure IMPC is infrequent, found in about 1%–2% of breast carcinomas. A threshold of 75% IMPC component is typically recommended to histologically classify a carcinoma as pure IMPC. Conversely, the presence of a micropapillary component in invasive carcinomas of no special type (NST) is more common, leading to substantial discussion regarding its clinical implications. Numerous studies have reported a correlation between the presence of the micropapillary feature in tumors and poorer prognosis, along with a pronounced lymphotropic pattern [4]. Epidemiological research indicates a consistent rise in the reported incidence of IMPC over recent decades, which may result from enhanced pathological awareness and refined histological examination techniques [5].

**Figure 1.**
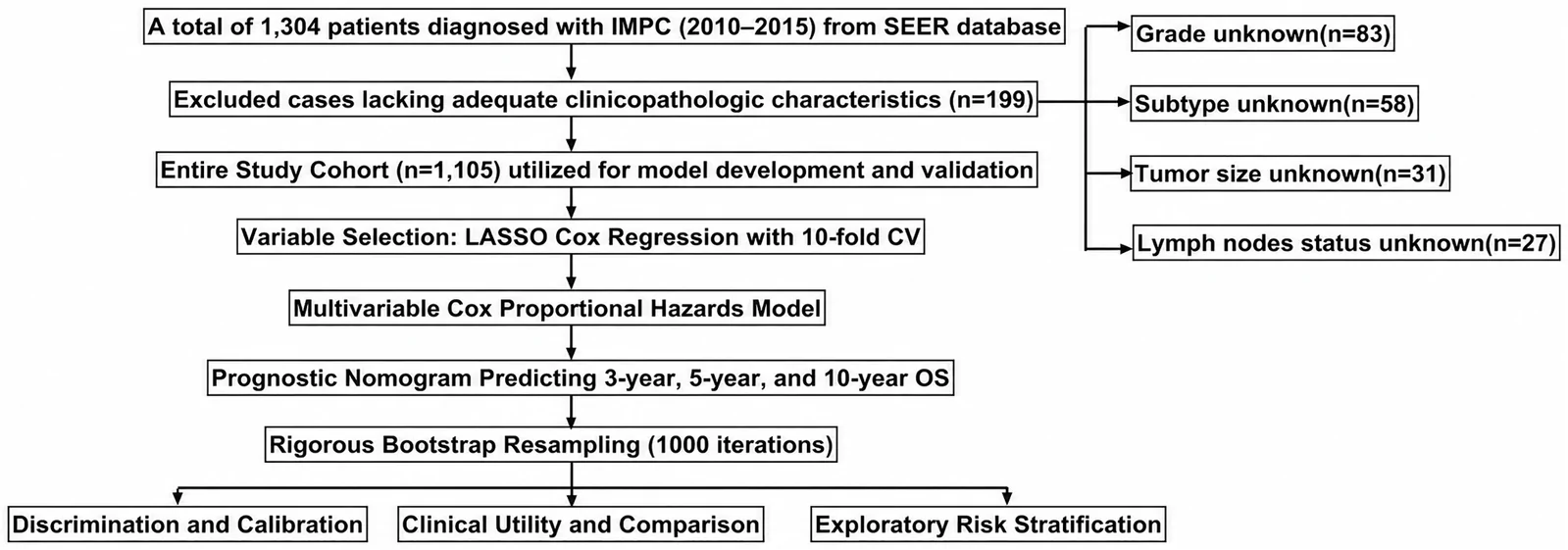
**Flowchart of the study design**. **Abbreviations:** LASSO: Least Absolute Shrinkage and Selection Operator; OS: Overall Survival; SEER: Surveillance, Epidemiology, and End Results.

Compared to invasive ductal carcinoma, not otherwise specified (IDC-NOS), the most distinctive feature of IMPC is the formation of tightly clustered tumor cells surrounded by clear stromal spaces, yielding a characteristic “micropapillary” architecture with a marked propensity for lymphatic invasion (lymphotropism) [6]. This aggressive biological behavior contributes to a high rate of lymph node metastasis at diagnosis, even in patients with relatively small primary tumors, leading to an unfavorable prognosis [7]. Currently, the diagnosis of IMPC primarily depends on histopathological evaluation, including the demonstration of reversed cell polarity through epithelial membrane antigen (EMA) immunostaining [8, 9]. Given its aggressive nature, IMPC typically necessitates more intensive treatment strategies [10]. Surgical resection remains the cornerstone of therapy, often followed by adjuvant chemotherapy, radiotherapy, and endocrine therapy when indicated [11]. However, the chemosensitivity of IMPC remains a subject of debate, and there is no consensus on the optimal comprehensive treatment strategy for this specific subtype [12]. Thus, due to the variability in treatment approaches and clinical outcomes, accurate prognostic assessment for patients with IMPC poses a significant clinical challenge.

For decades, the tumor–node–metastasis (TNM) staging system established by the American Joint Committee on Cancer (AJCC) has been regarded as the gold standard for prognostic evaluation in malignant tumors [13]. Nevertheless, TNM staging primarily focuses on anatomical extent, categorizing patients into broad risk groups without adequately considering individual patient characteristics (such as age, race, and marital status) or the conditional relationship of therapeutic interventions (surgery, chemotherapy, and radiotherapy) with survival outcomes. Furthermore, in IMPC—where lymph node metastasis is particularly prevalent—the conventional N stage, determined solely by the number of positive lymph nodes, is vulnerable to “stage migration” (the Will Rogers phenomenon) due to variability in the total number of examined lymph nodes, potentially failing to accurately reflect the true lymphatic tumor burden [14]. In this context, the log odds of positive lymph nodes (LODDS) has emerged as a novel lymph node staging metric, demonstrating superior prognostic performance compared to traditional N staging across various malignancies; however, its application in IMPC has been infrequently explored.

Among existing prognostic tools, nomograms are recognized as high-precision, user-friendly models that integrate multiple prognostic variables to provide individualized survival predictions for cancer patients. In contrast to the AJCC staging system, nomograms offer refined prognostic estimates by incorporating treatment-related variables and detailed pathological characteristics. Although a limited number of studies have sought to develop prognostic models for IMPC, many have been constrained by small sample sizes and often overlook detailed treatment information (such as surgical procedures and adjuvant therapies) or fail to include novel lymph node indicators like LODDS. To date, no IMPC-specific nomogram that comprehensively integrates treatment data and LODDS based on a large population has been reported. Therefore, this study aims to develop and validate a novel nomogram that incorporates treatment-related variables using data from the Surveillance, Epidemiology, and End Results (SEER) database to predict overall survival (OS) in patients with IMPC, while comparing its prognostic performance against the conventional AJCC staging system, ultimately providing a more robust tool for clinical decision-making.

## Materials and methods

### Data source and study population

The flowchart of this study is illustrated in Figure 1. Data were obtained from the SEER database maintained by the U.S. National Cancer Institute (NCI). Patients diagnosed with IMPC between 2010 and 2015 were identified using SEER*Stat software (version 8.3.9) from the SEER-8 registry database (1975–2022). Case identification was based on the International Classification of Diseases for Oncology, Third Edition (ICD-O-3), utilizing both histology and primary site codes. All data were extracted using SEER*Stat. Inclusion criteria included: (1) pathologically confirmed IMPC of the breast (ICD-O-3 code: 8507/3); (2) diagnosis year between 2010 and 2015; (3) primary tumor only; and (4) age > 18 years at diagnosis. Exclusion criteria were: (1) diagnosis based solely on autopsy or death certificate; (2) follow-up duration of less than 1 month; and (3) missing essential clinical or pathological information. As the SEER database is publicly available and contains de-identified data, this study was exempt from additional institutional review board approval.

### Variable definition and processing

Clinicopathological variables were retrospectively extracted from the SEER database. Collected variables included demographic characteristics (age, race, marital status, income, and rural–urban continuum code), tumor characteristics (laterality, primary tumor site, histological grade, tumor size, molecular subtype, and T, N, and M stages), and treatment modalities.

Molecular subtypes were classified into four categories based on estrogen receptor (ER), progesterone receptor (PR), and human epidermal growth factor receptor 2 (HER2) status: luminal A, luminal B, HER2-enriched, and triple-negative breast cancer (TNBC). TNM staging was uniformly defined according to the AJCC staging manual.

The LODDS was introduced as a novel prognostic indicator to evaluate lymph node burden. LODDS was calculated using the formula: LODDS = log((pNODES + 0.5) / (tNODES − pNODES + 0.5)), where pNODES represents the number of positive lymph nodes and tNODES represents the total number of examined lymph nodes. The addition of 0.5 to both the numerator and denominator avoids computational singularities when the number of positive or negative lymph nodes is zero. Higher LODDS values indicate a greater burden of lymph node metastasis.

Prior to model construction, restricted cubic splines (RCS) with three knots were applied to assess the linearity assumptions of continuous variables, including age, tumor size, and LODDS. RCS analysis demonstrated that these continuous variables exhibited linear relationships with the log relative hazard (*P* for non-linearity > 0.05; Supplementary Figure S1). Consequently, to maintain model interpretability and facilitate nomogram construction, these variables were incorporated into the final Cox models as linear continuous variables. Other categorical variables (e.g., race, histological grade, and treatment modalities) were treated as factor variables. Cases with missing values in key prognostic variables were excluded to ensure data quality. Cases classified as ‘unknown’ or ‘blank’ for surgical intervention, chemotherapy, or radiotherapy were considered missing and subsequently excluded.

The primary endpoint of this study was OS, defined as the interval from the date of diagnosis to death from any cause or the date of last follow-up, measured in months. Patients who were alive at the last follow-up were censored at that time.

### Ethical statement

This study utilized data from the SEER database. Given that the database contains publicly available, de-identified patient information, this study was exempt from formal institutional review board (IRB) approval and the requirement for individual informed consent, in accordance with the SEER data use agreement and relevant institutional policies.

**Table 1:** Baseline clinicopathologic characteristics of patients with invasive micropapillary carcinoma of the breast in the study cohort (*n* ═ 1105).

**Variables**	**Total (*n* ═ 1105)**
**Age**, mean ± SD	61.29 ± 13.21
**Tumor size**, mean ± SD	23.30 ± 19.04
**Survival months**, mean ± SD	97.99 ± 33.05
**LODDS**, mean ± SD	−0.82 ± 1.01
**Marital status**, *n* (%)	
Unmarried	516 (46.70)
Married	589 (53.30)
**Race**, *n* (%)	
Black	125 (11.31)
White	850 (76.92)
Other	130 (11.76)
**Laterality**, *n* (%)	
Left	537 (48.60)
Right	568 (51.40)
**Location**, *n* (%)	
Upper-inner	162 (14.66)
Lower-inner	89 (8.05)
Upper-outer	315 (28.51)
Lower-outer	86 (7.78)
Other	453 (41.00)
**Grade**, *n* (%)	
I	85 (7.69)
II	647 (58.55)
III	373 (33.76)
**T Stage**, *n* (%)	
T1	649 (58.73)
T2	349 (31.58)
T3	73 (6.61)
T4	34 (3.08)
**N Stage**, *n* (%)	
N0	598 (54.12)
N1	322 (29.14)
N2	107 (9.68)
N3	78 (7.06)
**M Stage**, *n* (%)	
M0	1078 (97.56)
M1	27 (2.44)
**OS**, *n* (%)	
Alive	842 (76.20)
Dead	263 (23.80)
**Income**, *n* (%)	
<69,999$	219 (19.82)
70,000∼79,999$	279 (25.25)
80,000∼94,999$	311 (28.14)
>95,000$	296 (26.79)
**Residence**, *n* (%)	
Rural	82 (7.42)
Urban	1023 (92.58)
**Subtype**, *n* (%)	
HR+	839 (75.93)
HER2+	41 (3.71)
HR−/HER2−	42 (3.80)
HR+/HER2+	183 (16.56)
**Surgery**, *n* (%)	
No surgery	34 (3.08)
BCS	567 (51.31)
Mastectomy	504 (45.61)
**Radiotherapy**, *n* (%)	
No	472 (42.71)
Yes	633 (57.29)
**Chemotherapy**, *n* (%)	
No	554 (50.14)
Yes	551 (49.86)

**Abbreviations:** SD: Standard deviation; LODDS: Log odds of positive lymph nodes; OS: Overall survival; HR: Hormone receptor; HER2: Human epidermal growth factor receptor 2; BCS: Breast-conserving surgery.

### Statistical analysis

#### Descriptive statistics and variable processing

Continuous variables are reported as mean ± standard deviation (SD) or median (interquartile range, IQR) as appropriate, while categorical variables are expressed as frequencies and percentages. All statistical analyses were performed following a prespecified analytic protocol using R software (R Foundation for Statistical Computing, Vienna, Austria; version 4.5.2). Two-sided *P*-values < 0.05 were considered statistically significant. Missing data were addressed using complete-case analysis. To optimize the use of available information and enhance statistical power, continuous variables such as age, tumor size, and the LODDS were retained on their original scales rather than being categorized. Prior to the construction of models, RCS with three knots at the 10th, 50th, and 90th percentiles were applied to evaluate the functional form and assess the linearity assumption in the Cox model. As no significant non-linear associations were detected (*P* for non-linearity > 0.05), these variables were modeled as linear continuous predictors.

#### Variable selection and model construction

To mitigate overfitting and address potential multicollinearity among high-dimensional clinicopathological features, variable selection was carried out using the least absolute shrinkage and selection operator (LASSO) Cox proportional hazards regression (via the ‘*glmnet*’ package). The optimal penalty parameter (λ) was determined through 10-fold cross-validation, applying the minimum mean cross-validated error criterion (lambda.min). The lambda.min criterion was intentionally selected over the 1 standard error criterion (lambda.1se) to avoid excessive penalization, thus ensuring that essential, clinically validated oncological variables (such as tumor stage and treatment modalities) were retained in the model alongside strict statistical regularization. Predictors with nonzero coefficients were subsequently incorporated into a multivariable Cox proportional hazards regression model to estimate adjusted hazard ratios (HRs) and 95% confidence intervals (CIs). Model assumptions were formally examined; the proportional hazards assumption was assessed using the global Schoenfeld residuals test, and variance inflation factors (VIFs) > 5 were considered indicative of substantial collinearity.

#### Model validation and comparison

To avoid information loss associated with traditional data splitting, the entire study cohort was employed for model development, augmented by rigorous bootstrap resampling techniques (1000 iterations) for internal validation. Model discrimination was initially quantified using the apparent Harrell’s Concordance index (C-index). Importantly, the entire modeling process—including LASSO feature selection and Cox regression—was repeated within each bootstrap sample to calculate the expected optimism, which was subsequently subtracted from the apparent performance to derive the optimism-corrected C-index. To ensure a strictly fair comparison, a baseline comparator model was constructed as a formal multivariable Cox proportional hazards regression utilizing the conventional 8th edition AJCC, T stage, N stage, and M stage as categorical covariates. This TNM-only comparator model was embedded within the same rigorous 1000-iteration bootstrap resampling pipeline as our proposed nomogram. All subsequent evaluations, including the calculation of apparent performance, expected optimism, optimism-corrected C-index, and calibration slopes, were executed identically for both models to guarantee an unbiased assessment. The difference in discrimination (ΔC-index) between the models was evaluated using Harrell’s paired U-statistic. Time-dependent receiver operating characteristic (ROC) curves with inverse probability of censoring weighting (IPCW) were employed to calculate the area under the curve (AUC).

Model calibration was initially assessed visually using calibration plots with 1,000 bootstrap iterations. Furthermore, to comprehensively evaluate the model’s predictive accuracy at specific clinical horizons (3, 5, and 10 years), we computed the time-dependent Brier score, calibration intercept (calibration-in-the-large), and calibration slope. To account for right-censoring without introducing bias, all horizon-specific calibration metrics were computed using IPCW. The Brier score was calculated as the IPCW-weighted mean squared difference between predicted risks and observed status. For the calibration intercept and slope, a generalized linear model was fitted to the actual event status against logit-transformed predicted risks, weighted by the inverse of the Kaplan-Meier estimate of the censoring distribution. A Brier score closer to 0 indicates lower prediction error, while a calibration intercept near 0 and a slope near 1 indicate perfect calibration.

**Figure 2.**
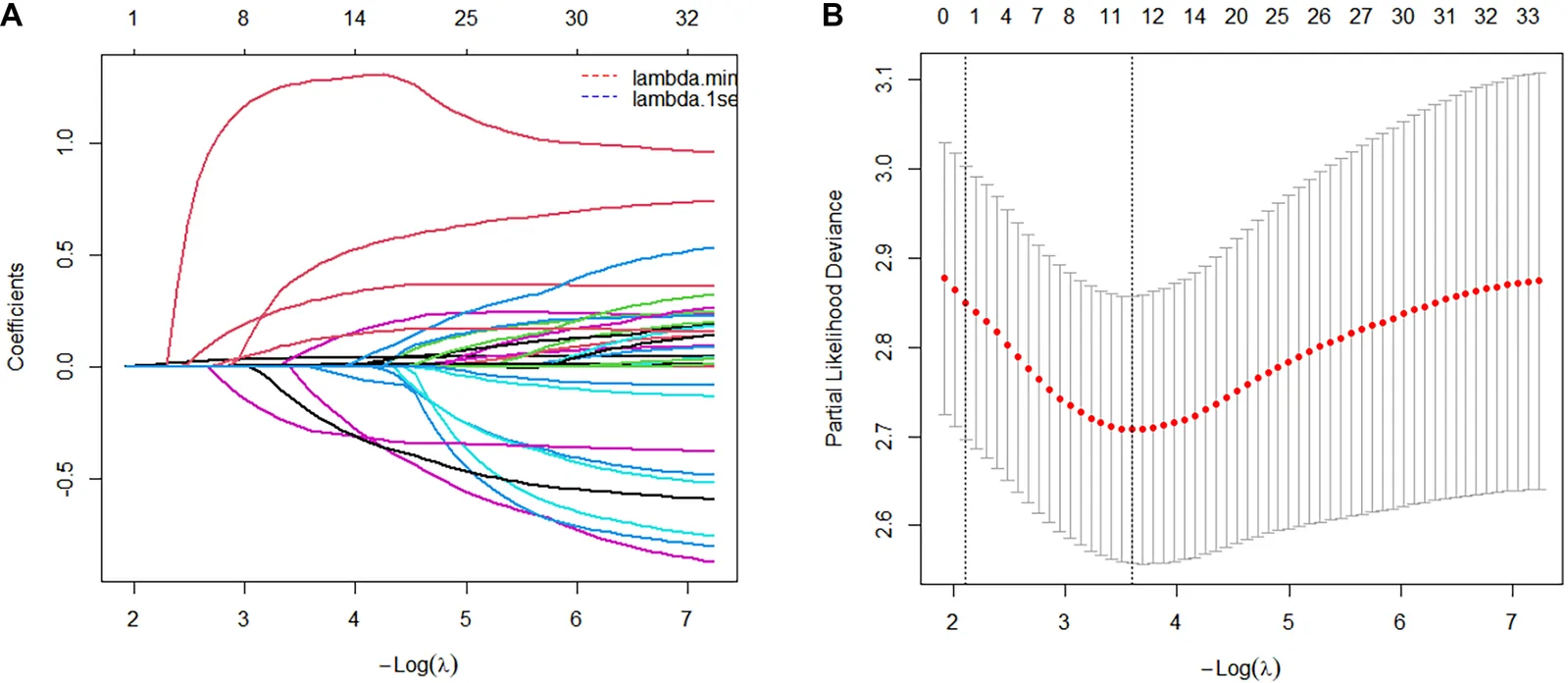
**Feature selection using the LASSO cox regression model.** (A) LASSO coefficient profiles for high-dimensional clinical features. Each curve illustrates the trajectory of a feature coefficient as the penalty parameter (λ) varies. As the penalty increases (moving from right to left along the x-axis), the coefficients are progressively shrunk toward zero. (B) Selection of the tuning parameter (λ) in the LASSO model via 10-fold cross-validation. The partial likelihood deviance (y-axis) is plotted against log(λ) (x-axis). Red dots indicate the mean deviance for each λ, while the error bars represent the corresponding confidence intervals. The two vertical dashed lines denote the optimal values derived from the minimum criteria (lambda.min, left dashed line) and the one standard error of the minimum criteria (lambda.1se, right dashed line). In this study, the optimal λ was chosen based on the lambda.min criterion to identify predictors with non-zero coefficients for the subsequent multivariable model. **Abbreviation:** LASSO: Least absolute shrinkage and selection operator.

To rigorously quantify the incremental predictive value and reclassification ability of the proposed model over the traditional TNM staging system, the continuous net reclassification improvement (NRI) and integrated discrimination improvement (IDI) were calculated using the ‘survIDINRI’ package with 500 bootstrap iterations. Instead of relying solely on statistical significance (*P* values) for reclassification, our interpretation focused on the point estimates and 95% confidence intervals of the IDI to emphasize the magnitude of absolute changes in predicted risks. Additionally, to evaluate clinical utility and net benefit across a continuum of decision-relevant thresholds without reliance on arbitrary risk categorizations, decision curve analysis (DCA) and clinical impact curves (CICs) were employed.

#### Clinical utility and risk stratification

To facilitate clinical application, regression coefficients from the final multivariable model were utilized to construct a nomogram for visual survival prediction. DCA was conducted with 1000 bootstrap resamples to assess the net clinical benefit of the nomogram across a range of threshold probabilities in comparison with the standard TNM staging system. Furthermore, CICs were plotted to visualize the estimated number of high-risk patients vs true positive cases. For clinical risk stratification, the optimal cutoff value of the nomogram-derived risk score was determined using the surv_cutpoint function. Patients were subsequently stratified into high- and low-risk groups, and differences in survival were compared using the Kaplan-Meier method and the log-rank test. A fixed random seed was set prior to all resampling procedures to ensure computational reproducibility. The final model formula, exact regression coefficients, and implementation details are provided in Table S1.

## Results

### Baseline characteristics of patients

Table 1 summarizes the baseline clinicopathological characteristics of the 1,105 patients included in this study. The mean age at diagnosis was 61.29 ± 13.21 years, with a mean tumor size of 23.30 ± 19.04 mm. The median follow-up duration was approximately 98 months (97.99 ± 33.05). The mean LODDS was −0.82 ± 1.01.

Most patients were White (76.92%) and married (53.30%), and the vast majority resided in urban areas (92.58%). Tumors were nearly evenly distributed between the left and right breasts, with the upper-outer quadrant being the most common primary location (28.51%).

Pathologically, grade II tumors predominated (58.55%), followed by grade III (33.76%). The cohort was largely diagnosed at an early stage, with 58.73% classified as T1 and 54.12% as N0; only 2.44% presented with distant metastasis.

HR-positive tumors accounted for the majority of cases (75.93%), whereas HER2-positive and triple-negative subtypes were relatively uncommon. Nearly all patients underwent surgery (96.92%), with breast-conserving surgery slightly more frequent than mastectomy. Approximately half of the patients received chemotherapy (49.86%), and 57.29% received radiotherapy.

Of the initial 1,304 patients identified, 199 were excluded due to missing clinicopathological data (detailed in Figure 1), yielding a final analytic cohort of 1105 patients.

### Screening for independent prognostic factors

In the entire cohort, LASSO Cox regression analysis was initially performed to screen candidate variables and prevent overfitting. The coefficient profiles of the candidate variables are shown in Figure 2A. The optimal penalization parameter (λ) was determined using 10-fold cross-validation, and the partial likelihood deviance reached its minimum value when log(λ) was optimal (Figure 2B). Based on the LASSO results, 10 variables with non-zero coefficients were identified as potential predictors, including Age, Marital Status, Grade, Tumor Size, M Stage, Rural-Urban Status, Subtype, Radiotherapy, Chemotherapy, and LODDS. These selected variables were subsequently entered into a multivariate Cox proportional hazards regression model. In the entire cohort, 263 events were observed for 10 candidate predictors, yielding an events-per-variable (EPV) ratio of approximately 26.3, which exceeds the commonly recommended minimum threshold and supports the stability and reliability of the regression estimates.

In the multivariable Cox proportional hazards regression analysis (Table 2), Marital Status, Age, Tumor Size, M Stage, LODDS, Chemotherapy, Residence, and Radiotherapy demonstrated statistical significance (*P <* 0.05). Notably, while Histological Grade did not achieve statistical significance (*P* > 0.05), only the HR-/HER2- category within the Molecular Subtype exhibited a significant association with survival (*P* ═ 0.011) in the multivariable model. Both variables were retained in the final nomogram due to their selection via the LASSO regression method, which emphasizes minimizing prediction error and preventing overfitting over individual variable significance. Additionally, these factors are clinically recognized as essential for breast cancer management, ensuring the comprehensive nature of the prognostic model. The proportional hazards assumption was validated by the Schoenfeld residuals test, which indicated no significant violation of the assumption for the final multivariable model (Global *P* ═ 0.082).

**Table 2:** Univariable and multivariable Cox proportional hazards regression analyses of overall survival.

	** Univariable analysis**			**Multivariable analysis**			
**Variables**	***n***	**HR**	**95% CI**	***P* value**	**HR**	**95% CI**	***P* value**
**Marital status**	1,105						
Unmarried		—	—		—	—	
Married		0.44	0.34, 0.56	<0.001	0.66	0.50, 0.85	0.002
**Race**	1,105						
Black		—	—				
White		0.64	0.46, 0.90	0.010			
Other		0.51	0.31, 0.84	0.009			
**Laterality**	1,105						
Left		—	—				
Right		0.99	0.78, 1.26	0.921			
**Location**	1,105						
Upper-inner		—	—				
Lower-inner		1.03	0.59, 1.80	0.923			
Upper-outer		1.21	0.81, 1.81	0.359			
Lower-outer		0.98	0.55, 1.75	0.938			
Other		1.32	0.90, 1.93	0.154			
**Grade**	1,105						
I		—	—		—	—	
II		0.97	0.60, 1.56	0.887	0.95	0.59, 1.54	0.845
III		1.32	0.81, 2.15	0.266	1.31	0.79, 2.18	0.295
**T stage**	1,105						
T1		—	—				
T2		1.32	1.01, 1.73	0.042			
T3		2.00	1.32, 3.02	0.001			
T4		3.88	2.34, 6.43	<0.001			
**N stage**	1,105						
N0		—	—				
N1		0.92	0.69, 1.23	0.563			
N2		1.10	0.72, 1.67	0.659			
N3		1.94	1.32, 2.86	<0.001			
**M stage**	1,105						
M0		—	—		—	—	
M1		6.62	4.19, 10.5	<0.001	3.77	2.26, 6.29	<0.001
**Income**	1,105						
<69,999$		—	—				
70,000∼79,999$		1.04	0.73, 1.49	0.824			
80,000∼94,999$		1.03	0.72, 1.46	0.878			
>95,000$		0.90	0.62, 1.29	0.555			
**Residence**	1,105						
Rural		—	—		—	—	
Urban		0.67	0.45, 0.99	0.046	0.57	0.38, 0.85	0.006
**Subtype**	1,105						
HR+		—	—		—	—	
HER2+		0.80	0.40, 1.63	0.544	1.04	0.50, 2.13	0.925
HR−/HER2−		2.50	1.58, 3.96	<0.001	1.93	1.16, 3.21	0.011
HR+/HER2+		0.78	0.54, 1.12	0.185	0.98	0.67, 1.45	0.933
**Surgery**	1,105						
No surgery		—	—				
BCS		0.13	0.08, 0.20	<0.001			
Mastectomy		0.17	0.11, 0.25	<0.001			
**Variables**	***n***	**HR**	**95% CI**	***P* value**	**HR**	**95% CI**	***P* value**
**Radiotherapy**	1,105						
No		—	—		—	—	
Yes		0.52	0.40, 0.66	<0.001	0.65	0.50, 0.83	<0.001
**Chemotherapy**	1,105						
No		—	—		—	—	
Yes		0.52	0.40, 0.67	<0.001	0.60	0.45, 0.81	<0.001
**Age**	1,105	1.06	1.05, 1.07	<0.001	1.05	1.03, 1.06	<0.001
**Tumor size**	1,105	1.01	1.01, 1.02	<0.001	1.01	1.01, 1.02	<0.001
**LODDS**	1,105	1.34	1.20, 1.49	<0.001	1.23	1.09, 1.38	<0.001

**Abbreviations:** HR: Hazard ratio; CI: Confidence interval.

To rigorously address potential confounding by indication regarding the included treatment modalities, we conducted a series of sensitivity analyses. These included a stratified Cox model adjusting for baseline hazard differences across treatments, a restricted cohort analysis involving only surgical patients, and subgroup analyses stratified by chemotherapy status. Across all sensitivity models, core prognosticators, particularly LODDS, retained their independent prognostic significance (all *P <* 0.05; Tables S2–S4). This confirms the structural stability of the selected features, irrespective of treatment biases.

### Construction and verification of the prognostic nomogram

Based on the independent prognostic factors identified in the multivariable Cox proportional hazards model, we constructed a prognostic nomogram to estimate the probability of 3-, 5-, and 10-year OS for patients with IMPC (Figure 3). Each predictor, including age, tumor size, LODDS, M stage, and other retained covariates, was assigned a weighted point value proportional to its regression coefficient. The total score for an individual patient was calculated by summing the points for all predictors, corresponding to the predicted survival probabilities indicated on the lower scales of the nomogram.

**Figure 3.**
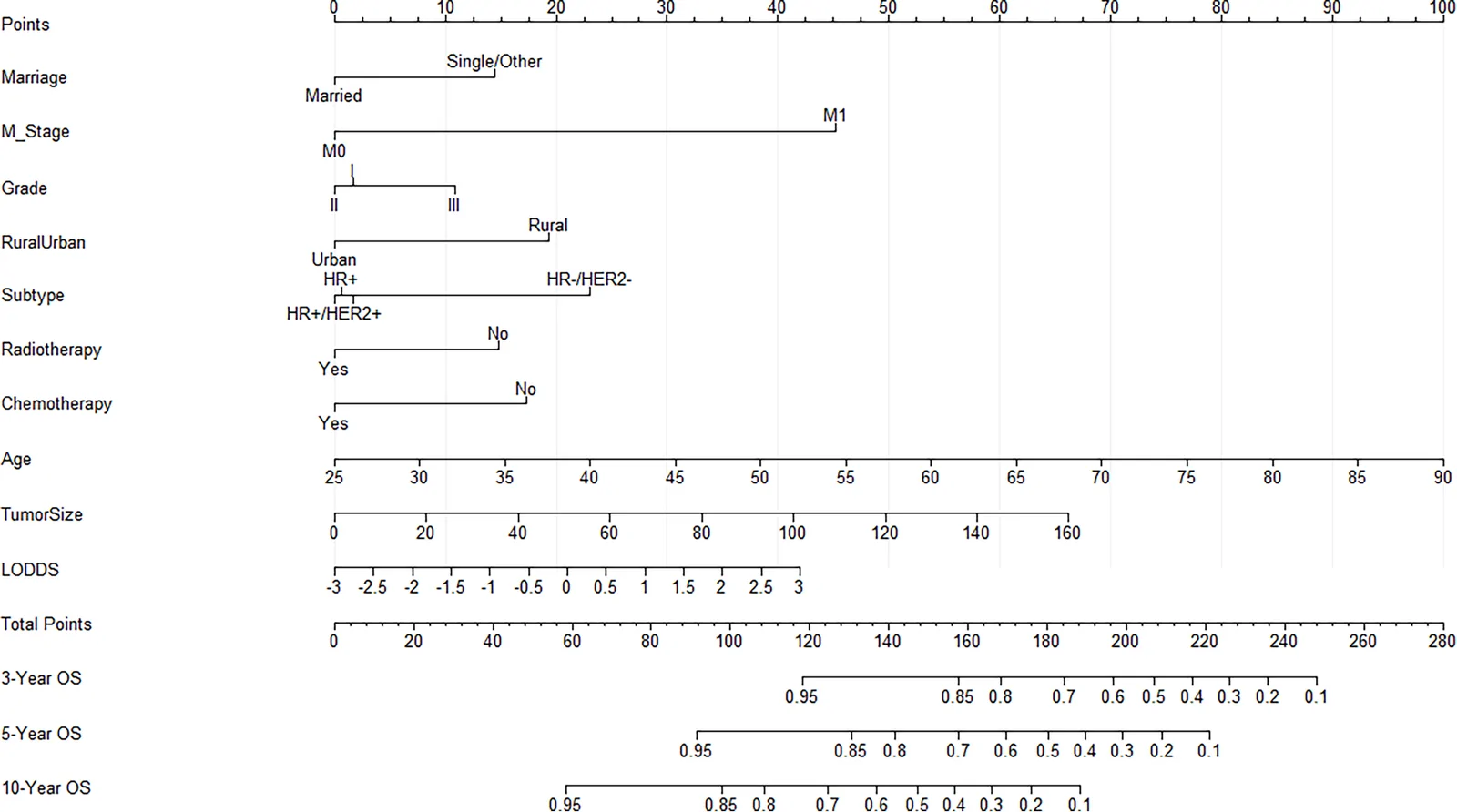
**Nomogram for predicting OS at 3, 5, and 10 years.** This nomogram was developed using ten independent prognostic factors identified through LASSO and multivariable Cox regression analyses. Instructions for using the nomogram: Identify the patient’s status or value on each corresponding variable axis. Draw a vertical line upward to the “Points” axis to determine the score for that variable. Sum the scores of all variables to calculate the “Total Points.” Finally, locate this sum on the “Total Points” axis and draw a vertical line downward to the survival axes to obtain the individualized estimated probabilities of overall survival at 3, 5, and 10 years. **Abbreviations:** OS: Overall survival; LASSO: Least absolute shrinkage and selection operator; LODDS: Log odds of positive lymph nodes.

The developed LASSO-Cox proportional hazards model (using the lambda.min criterion) demonstrated strong discriminative ability, yielding an apparent Harrell’s C-index of 0.761. To rigorously assess the model’s internal validity and correct for potential overfitting induced by the high-dimensional variable selection process, we performed a bootstrap validation procedure with 1,000 resamples. The optimism-corrected C-index remained robust at 0.738, with a minimal mean optimism of 0.022. These results indicate that the prediction model maintains stable performance and a low degree of overfitting, ensuring reliable generalization to future patient populations.

To ensure a fair comparison, we constructed a baseline Cox proportional hazards model utilizing only the conventional TNM staging system and evaluated it using the identical 1,000-iteration bootstrap optimism-correction procedure. The apparent C-index of the TNM model was 0.616, which decreased to an optimism-corrected C-index of 0.581 (mean optimism = 0.035). In contrast, our proposed LASSO-Cox model maintained a robust optimism-corrected C-index of 0.738. The paired U-statistic test revealed a statistically significant improvement in discriminative ability for the proposed model over the conventional TNM system, with a C-index difference of 0.157 (95%CI: 0.1096–0.1864; *P <* 0.001). This rigorously demonstrates the superior and independent prognostic value of the features identified through LASSO regularization. Additionally, time-dependent ROC curves visually and quantitatively corroborated this superiority at specific time points (Figure 4). The nomogram exhibited consistently higher AUC values for predicting 3-, 5-, and 10-year OS compared to the conventional TNM staging system (3-year: 0.812 vs 0.676, *P* ═ 0.001; 5-year: 0.792 vs. 0.653, *P <* 0.001; 10-year: 0.798 vs. 0.606, *P <* 0.001). These results provide compelling evidence of the enhanced prognostic accuracy of the developed model across short- to long-term survival horizons.

**Figure 4.**
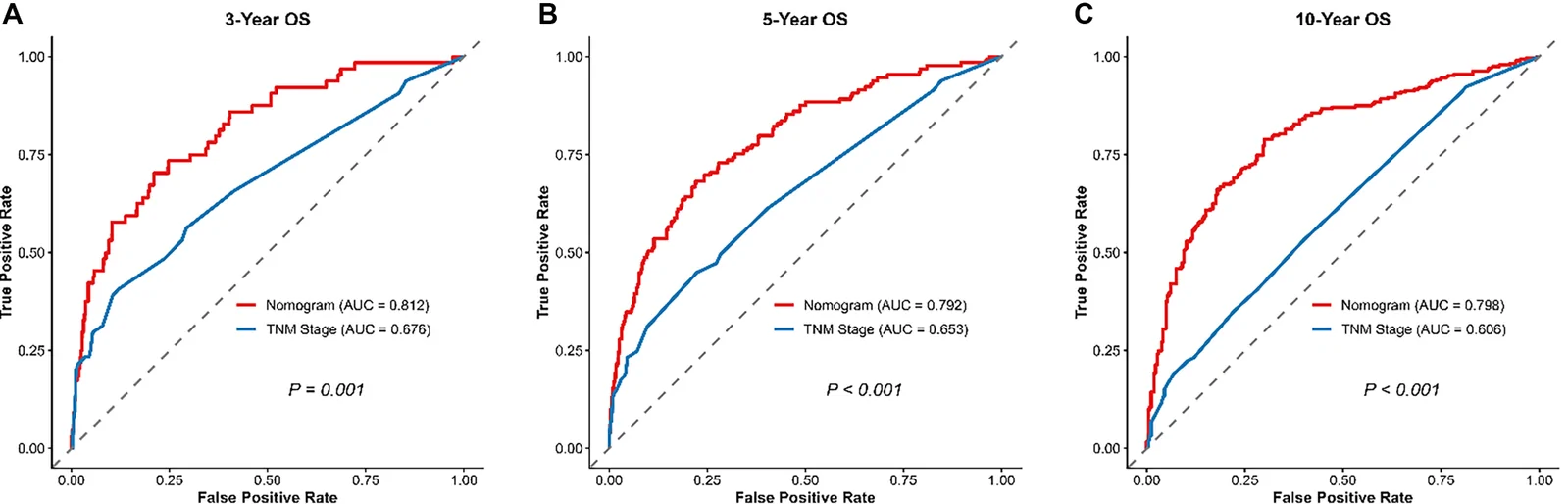
**Comparison of time-dependent ROC curves between the nomogram and the traditional TNM staging system.** The prognostic discriminative abilities for OS were assessed at (A) 3-year, (B) 5-year, and (C) 10-year intervals. The red curves illustrate the nomogram, while the blue curves depict the conventional TNM staging system. The AUC values and the statistical significance (*P* values) of the differences between the two models are indicated within each plot. At all assessed time points, the nomogram exhibited significantly greater predictive accuracy compared to the TNM staging system (all *P <* 0.05). **Abbreviations:** ROC: Receiver operating characteristic; TNM: Tumor–node–metastasis; OS: Overall survival; AUC: Area under the curve.

In addition to discrimination, the calibration of the nomogram was rigorously evaluated out-of-sample using the bootstrap resampling approach to account for optimism. Calibration plots visually demonstrated excellent agreement between predicted and observed OS probabilities at 3, 5, and 10 years (Figure 5). The overall optimism-corrected calibration slope was 0.9038 (95% CI: 0.6437–1.1184), indicating alignment between the predicted and observed survival probabilities without severe overfitting. Furthermore, horizon-specific calibration was assessed at 3, 5, and 10 years. The optimism-corrected mean absolute errors in predicted probabilities were exceptionally low at 0.0006, 0.0020, and 0.0034 for the 3-, 5-, and 10-year OS, respectively. To rigorously address the right-censored nature of the data, time-dependent Brier scores and additional calibration metrics were computed using the inverse probability of censoring weighting (IPCW) method. The IPCW-adjusted Brier scores were remarkably low at 0.049, 0.087, and 0.149 for the 3-, 5-, and 10-year horizons. Moreover, the IPCW calibration intercepts (0.280, 0.115, and 0.063) and slopes (1.136, 1.084, and 1.019) at these respective time points progressively approached the ideal values of 0 and 1, demonstrating excellent long-term predictive accuracy. Detailed calibration performance metrics are summarized in Table S5.

**Figure 5.**
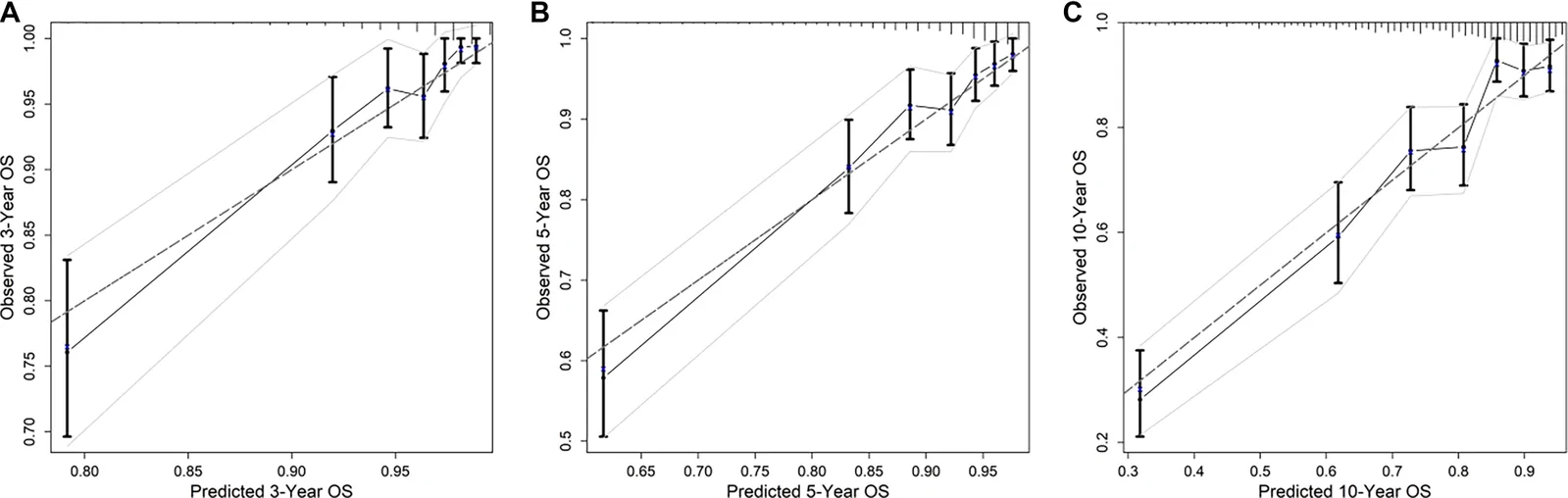
**Calibration plots of the nomogram for predicting OS.** Calibration plots illustrate the correspondence between predicted and observed OS probabilities at (A) 3 years, (B) 5 years, and (C) 10 years. The x-axis denotes the predicted survival probability derived from the nomogram, while the y-axis displays the bootstrap-corrected observed survival, estimated using the Kaplan–Meier method. The dashed 45∘ line signifies perfect calibration, whereas the solid line represents the bias-corrected calibration curve based on 1,000 bootstrap resamples. Vertical bars indicate 95% confidence intervals. The strong agreement between predicted and observed survival probabilities reflects the model’s robust calibration performance. **Abbreviation:** OS: Overall survival.

### Clinical utility and decision curve analysis

The clinical utility of the nomogram was evaluated using DCA, which compares the net benefit of various decision-making strategies across a range of threshold probabilities. As shown in Figure 6, the DCA curves for 3-, 5-, and 10-year OS demonstrated that the nomogram (teal line) consistently yielded a higher net benefit compared to the traditional TNM staging system (purple line), as well as the “treat-all” and “treat-none” strategies. Notably, for 10-year survival prediction, the nomogram showed a substantial improvement in net benefit over the TNM staging system across a wide threshold range at all three evaluated time points. This indicates that utilizing the proposed nomogram to guide clinical decision-making can provide greater survival benefits for patients than relying solely on the traditional TNM staging system. Additionally, to further assess clinical applicability, CICs were plotted for each time horizon (Figure 7). The results indicated that the nomogram possessed excellent risk stratification ability with a high true-positive rate, further supporting its clinical value.

**Figure 6.**
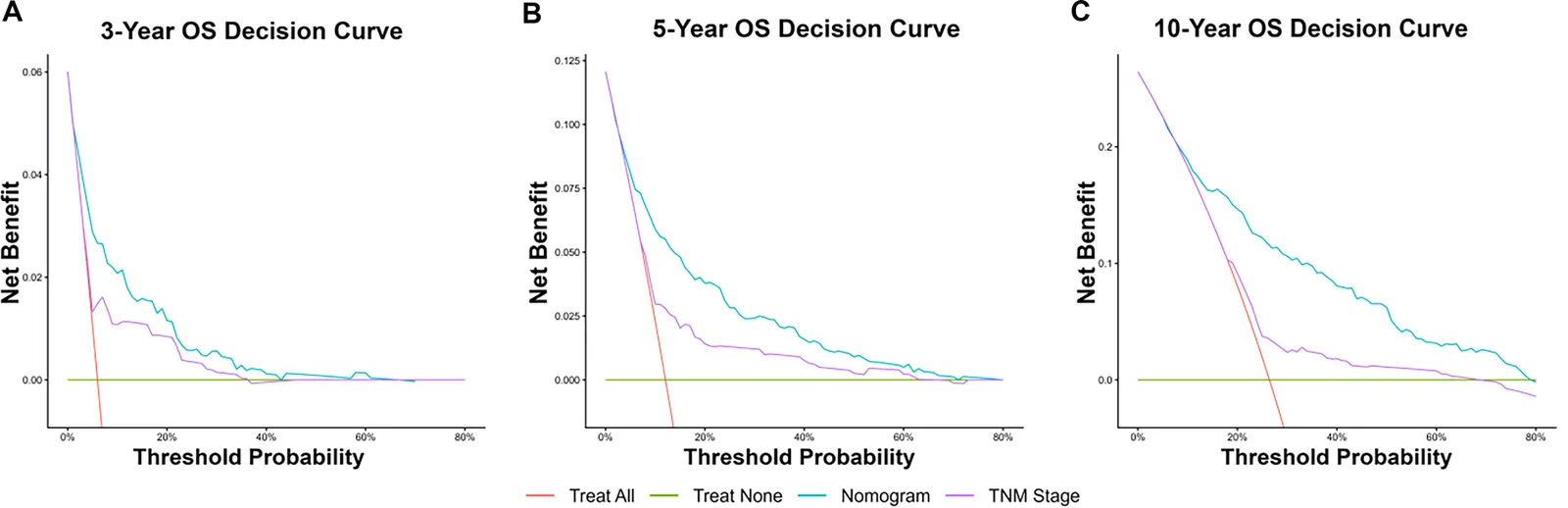
**DCA assessing the clinical utility of the nomogram versus the traditional TNM staging system for predicting OS.** (A) 3-year OS. (B) 5-year OS. (C) 10-year OS. The x-axis illustrates the threshold probability, while the y-axis denotes the net benefit. The green horizontal line (Treat None) indicates a scenario where no patients experience the event, resulting in a net benefit of zero. Conversely, the red downward-sloping line (Treat All) represents a situation where all patients encounter the event and receive intervention. The teal and purple lines depict the clinical net benefits of the developed nomogram and the traditional TNM staging system, respectively. Across all observed time points, the nomogram consistently exhibited a higher net benefit compared to both the TNM staging system and the baseline “Treat All” or “Treat None” strategies over a broad range of threshold probabilities, underscoring its superior clinical applicability. **Abbreviations:** DCA: Decision curve analysis; TNM: Tumor–node–metastasis; OS: Overall survival.

**Figure 7.**
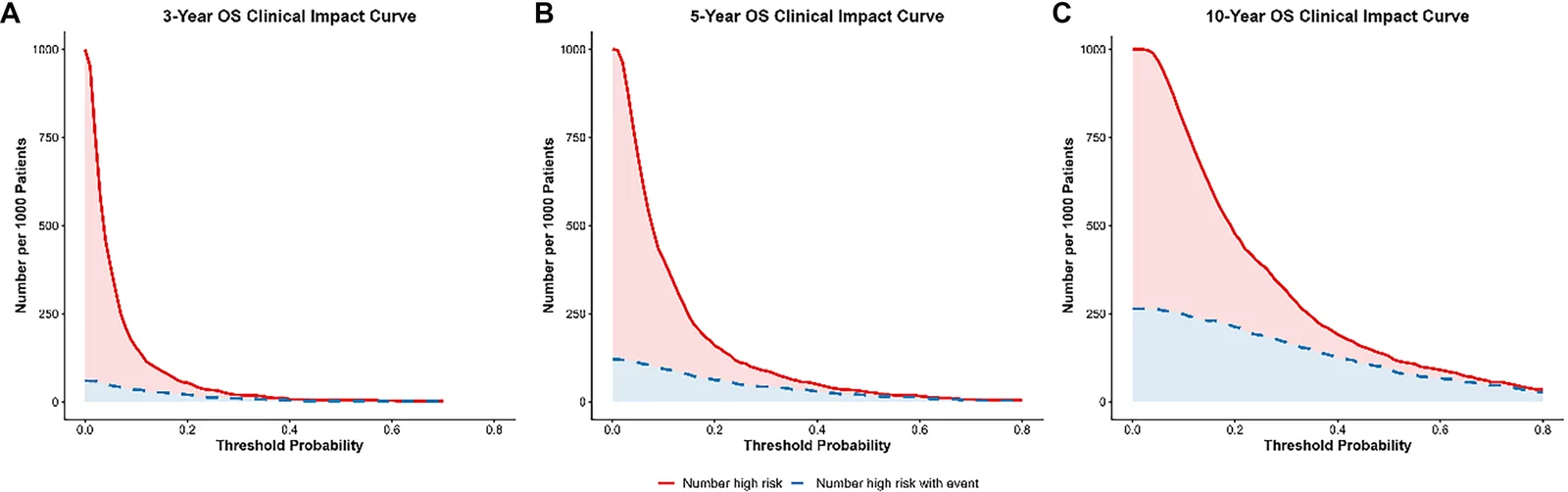
**CICs of the nomogram for predicting OS**. (A) 3-year OS. (B) 5-year OS. (C) 10-year OS. The horizontal axis represents the threshold probability, while the vertical axis indicates the number of patients per 1,000. The red solid line (“Number at High Risk”) illustrates the estimated number of patients classified as high risk by the nomogram at each threshold probability. The blue dashed line (“Number at High Risk with Event”) represents true positive cases, specifically the number of high-risk patients who actually experienced the event. The red shaded area between the two curves signifies false-positive cases (overtreatment), whereas the blue shaded area denotes true positives. The close alignment of the two curves, particularly at higher threshold probabilities, underscores the model’s excellent clinical utility, demonstrating a remarkably low incidence of overtreatment. **Abbreviations:** CICs: Clinical impact curves; OS: Overall survival.

### Reclassification and discriminative improvement

The incorporation of the selected variables into the TNM staging system significantly improved risk reclassification capabilities. To cautiously interpret the model’s utility and emphasize absolute risk changes, we focused on the IDI point estimates. At the 5-year horizon, the IDI was 0.108 (95% CI: 0.065–0.169), alongside a continuous NRI of 0.367 (95% CI: 0.250–0.456). This IDI indicates a clinically meaningful absolute risk change, suggesting that the new nomogram increased the average predicted probability gap between patients who experienced the event and those who did not by approximately 10.8%.

Similar substantial absolute improvements were observed at the 3-year horizon (Table S6), with an IDI of 0.077 (95% CI: 0.033–0.137; translating to a 7.7% absolute change gap), and an NRI of 0.363 (95% CI: 0.189–0.487). Furthermore, at the 10-year horizon, the model exhibited exceptional reclassification ability, achieving an IDI of 0.184 (95% CI: 0.129–0.237, reflecting an 18.4% absolute gap) and an NRI of 0.427 (95% CI: 0.338–0.514). Finally, DCA confirmed that the nomogram provided a consistently higher net benefit across a wide range of decision-relevant threshold probabilities compared to the traditional TNM system, thereby addressing clinical actionability beyond mere statistical reclassification.

### Risk stratification

To explore the potential clinical utility of the nomogram, patients were stratified into high- and low-risk groups based on their total risk scores. An optimal cutoff value of −0.2550 was determined in the entire study cohort using maximally selected rank statistics (via the surv_cutpoint function). Based on this threshold, 920 patients were classified into the low-risk group, while 185 patients were classified into the high-risk group in the entire cohort. Kaplan–Meier analysis revealed a clear separation in OS between the two groups, with the high-risk group exhibiting significantly poorer survival compared to the low-risk group (log-rank *P <* 0.0001; Figure 8).

**Figure 8.**
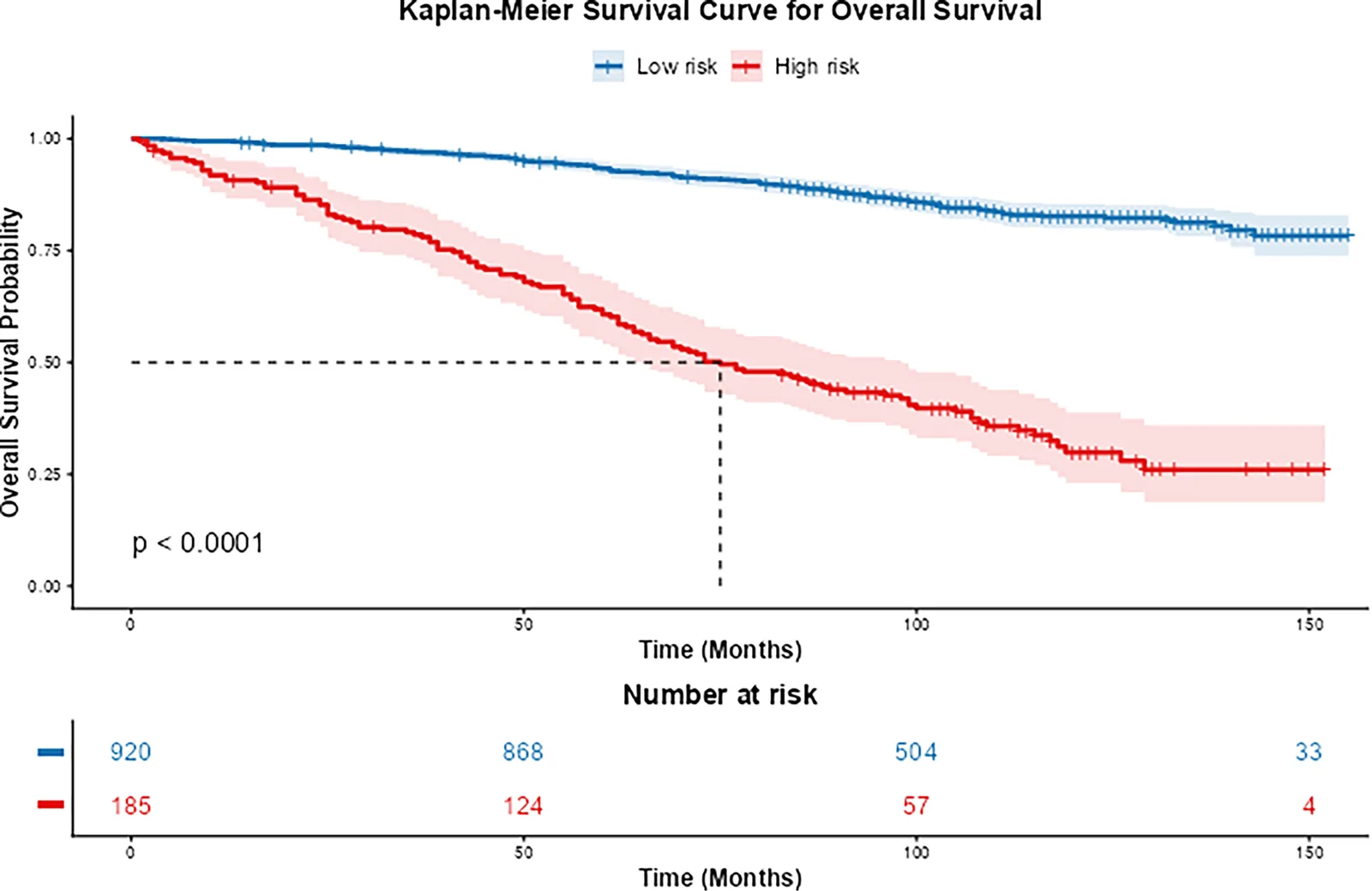
**Kaplan-Meier survival analysis based on nomogram-derived risk scores.** In this study cohort, patients were categorized into high-risk (red line) and low-risk (blue line) groups according to the optimal cutoff value derived from maximally selected rank statistics (surv_cutpoint). The high-risk group demonstrated significantly poorer OS compared to the low-risk group. The shaded areas indicate the 95% confidence intervals, and the statistical difference between the two survival curves was assessed using the log-rank test (*P <* 0.001). The table beneath the main plot presents the number of patients at risk at each designated time point. **Abbreviation:** OS: Overall survival.

However, since this optimal cutoff (−0.2550) was strictly data-driven, this risk stratification should be considered entirely exploratory. It is well recognized that optimal cutpoint selection inherently inflates Kaplan–Meier curve separation. Given that our study relies on internal validation, these findings primarily illustrate the model’s discriminative potential within the current dataset rather than establishing a definitive clinical threshold. The true clinical applicability of this specific cutpoint requires external validation in independent cohorts.

## Discussion

In this study, we conducted a comprehensive analysis of IMPC utilizing large-scale data from the SEER program. We developed an innovative, practical, and comprehensive nomogram that integrates key treatment-related variables—specifically chemotherapy and radiotherapy—and a novel lymph node staging metric, the LODDS, to estimate OS at 3, 5, and 10 years for patients with IMPC. The nomogram exhibited satisfactory discrimination and calibration across the entire study cohort. Compared to the conventional AJCC TNM staging system, our model demonstrated superior performance in terms of the C-index and DCA. While this nomogram indicates potential prognostic risk gradients, the specific cut points used for risk stratification are entirely data-driven, serving strictly exploratory purposes. Therefore, these stratifications should not be recommended as definitive clinical thresholds without rigorous external validation [15, 16].

Nomograms are increasingly recognized as valuable prognostic tools because they integrate multiple parameters—particularly treatment-related information—to provide quantitative, individualized survival estimates. Consequently, their predictive accuracy often surpasses that of anatomy-based AJCC staging systems, making them potential alternatives or complements to traditional staging approaches [17]. Although previous studies have explored prognostic factors in IMPC, most models either failed to comprehensively incorporate multidisciplinary treatment data or were limited to conventional N staging alone. Given the pronounced lymphophilic nature of IMPC and its strong propensity for early lymph node metastasis [18], anatomical staging alone may be insufficient for guiding complex clinical decision-making.

Surgery remains the cornerstone of IMPC management. In our univariable analysis, surgical intervention was significantly associated with a decreased mortality risk (*P <* 0.001), consistent with current clinical consensus that adequate local control is critical for this aggressive subtype. Although surgery was not retained as a significant independent predictor in the final multivariable model—likely due to the vast majority of the cohort (96.92%) undergoing surgical resection, limiting statistical variance—its fundamental necessity and survival benefit in clinical practice are undisputed. Moreover, given IMPC’s marked lymphophilic nature and high propensity for early lymph node metastasis, precise axillary staging is paramount. In this context, the emerging role of indocyanine green (ICG) as a sentinel lymph node tracer has shown significant promise in micropapillary breast carcinoma, potentially enhancing axillary evaluation and surgical planning to complement advanced staging metrics such as LODDS [19].

The roles of chemotherapy and radiotherapy in IMPC remain contentious in the literature. Some studies suggest that IMPC may exhibit lower sensitivity to chemotherapy compared to IDC-NOS. In our analysis, we observed a conditional association between the receipt of chemotherapy/radiotherapy and survival outcomes after adjusting for relevant clinicopathological covariates. These findings indicate a statistically significant relationship between adjuvant therapies and survival outcomes within our multivariable model. By integrating treatment variables, our nomogram facilitates a more objective assessment of the contribution of different therapeutic strategies to individual survival outcomes.

Additionally, the specific molecular characteristics of IMPC merit further examination. For instance, evaluating the clinical significance of HER2 expression in micropapillary breast carcinoma vs ductal carcinoma in situ (DCIS) provides crucial insights into tumor progression and aggressiveness, potentially refining individualized targeted therapy and prognostic stratification [20]. Furthermore, the favorable prognostic impact of marital status has been reported across various malignancies, possibly reflecting enhanced social support, improved psychological well-being, and better treatment adherence [21]. Other studies have explored associations between race, psychological and biological stress, and breast cancer outcomes [22], underscoring the intricate role of psychosocial factors in cancer progression.

A key innovation of this study is the integration of treatment data and LODDS, demonstrating its superiority over traditional N staging in IMPC. During multivariable model selection, LODDS emerged as an independent prognostic factor, supplanting N stage. Conventional N staging is constrained by its reliance on the total number of dissected lymph nodes and is susceptible to the Will Rogers phenomenon, which can lead to biased prognostic assessments due to stage migration. In contrast, LODDS concurrently accounts for both positive and negative lymph nodes, providing a more accurate representation of true lymph node burden [23]. Our results indicated that higher LODDS values correlate with worse survival outcomes, suggesting that LODDS should be emphasized in future IMPC staging and prognostic evaluations, particularly in cases with an inadequate number of examined lymph nodes [24].

Moreover, our nomogram outperformed the AJCC TNM staging system in predicting OS. While our model exhibited superior statistical reclassification abilities, we recognize that metrics such as continuous NRI must be interpreted with caution. Continuous NRI evaluates directional changes in predicted probabilities, which may sometimes reward infinitesimally small variations that do not necessarily influence clinical decisions. Given that universally accepted decision-analytic risk cutoffs for this specific patient cohort are not yet firmly established, defining strict categorical thresholds *a priori* may be arbitrary. Consequently, we emphasized the absolute risk changes indicated by the IDI—which revealed a significant absolute risk gap increase of up to 18.4% at 10 years, rather than relying solely on reclassification *P*-values. Notably, DCA demonstrated that for both 5- and 10-year survival predictions, the nomogram provided a higher net clinical benefit than the AJCC staging system and the “treat-all” or “treat-none” strategies across a wide range of threshold probabilities. The consistent net benefit observed across diverse decision-relevant thresholds in DCA, combined with clinically meaningful IDI results, collectively reaffirm the clinical utility of our nomogram beyond mere statistical reclassification. These findings suggest that clinical decision-making guided by this nomogram—such as identifying high-risk patients for intensified surveillance or treatment—may lead to improved clinical outcomes compared to reliance on TNM staging alone. The observed advantage in long-term (10-year) survival prediction is particularly pertinent for breast cancer, a disease characterized by substantial heterogeneity in long-term outcomes.

Several limitations of this study warrant acknowledgment. First, the nomogram was developed using retrospective data from the SEER database, which may introduce unavoidable selection and information biases, including the lack of detailed information on chemotherapy regimens, dosages, and targeted therapies. Second, the SEER database does not provide data on critical prognostic biomarkers such as the Ki-67 index, lymphovascular invasion (LVI), or genomic assay results (e.g., Oncotype DX) [25], which may limit the upper bound of predictive performance. Third, our evaluation of surgical interventions was restricted to broad categories of procedures (breast-conserving surgery vs mastectomy). Crucial granular surgical pathology details from resection specimens—such as precise margin status and the concurrent presence of ductal or lobular carcinoma in situ (DCIS/LCIS)—are not uniformly or comprehensively recorded in the SEER database. Consequently, we could not incorporate these significant prognostic variables into our nomogram, potentially limiting the model’s ability to fully capture the nuances of local surgical control. Fourth, our primary endpoint was OS, which inherently combines cancer-specific mortality with non-cancer-related deaths. Thus, the prognostic impact of tumor-specific variables (such as LODDS or tumor size) might be partially diluted by competing risks, particularly cardiovascular disease or aging-related mortality in long-term follow-ups. While OS is an objective endpoint free from cause-of-death misclassification bias, future studies focusing on breast cancer-specific survival (BCSS) with competing risk models are warranted to refine these predictions. Additionally, the observed survival benefits associated with treatment may be influenced by selection bias, as patients receiving more aggressive therapies generally have better baseline performance status. We acknowledge that the apparent survival association related to chemotherapy in our model might stem from confounding by indication and selection bias, as patients undergoing chemotherapy typically possess a better baseline performance status and specific clinicopathological risk profiles. Therefore, the predictive weight of treatment variables in our nomogram reflects conditional statistical associations rather than definitive causal effects and should be interpreted with caution. Although internal validation was performed, it is essential to recognize that internal validity does not guarantee external transportability. The predictive performance and clinical utility of this model may vary across different healthcare systems, geographic regions, or demographic groups. Thus, the proposed nomogram should be viewed as a preliminary prognostic tool. Further rigorous validation in independent, prospective external cohorts is necessary before it can be reliably recommended for routine clinical implementation. The missing data were managed through complete-case analysis, which may introduce slight bias compared to multiple imputation techniques, although the proportion of missing key variables was relatively small. Nonetheless, our extensive sensitivity analyses (Tables S2–S4) demonstrated that even after accounting for treatment-related indication biases through stratification, cohort restriction, and subgrouping, the robust prognostic utility of LODDS remained statistically significant, underscoring that our model’s discriminative ability is not merely a statistical artifact of treatment heterogeneity.

## Conclusion

We developed and internally validated a novel nomogram incorporating treatment variables and LODDS for patients with IMPC. Although it demonstrated promising discrimination and calibration, it should be considered a preliminary tool. External validation is required to confirm its transportability and clinical utility prior to widespread implementation.

## Supplemental data

Supplemental data are available at the following link: https://www.bjbms.org/ojs/index.php/bjbms/article/view/13884/4153.

## Data Availability

The datasets analyzed during the current study are available in the Surveillance, Epidemiology, and End Results (SEER) Program database (www.seer.cancer.gov). Access to these datasets requires the submission of a research proposal and the signing of the SEER data-use agreement. The processed data supporting the conclusions of this article are available from the corresponding author upon reasonable request.
